# Matcha Green Tea Powder does not Prevent Diet‐Induced Arteriosclerosis in New Zealand White Rabbits Due to Impaired Reverse Cholesterol Transport

**DOI:** 10.1002/mnfr.202100371

**Published:** 2021-08-27

**Authors:** Monika Hunjadi, Claudia Sieder, Anja Beierfuß, Christian Kremser, Bernhard Moriggl, René Welte, Christine Kastner, Demissew Shenegelegn Mern, Andreas Ritsch

**Affiliations:** ^1^ Department of Internal Medicine Medical University of Innsbruck Innsbruck Austria; ^2^ Central Laboratory Animal Facility Medical University of Innsbruck Innsbruck Austria; ^3^ Department of Radiology Medical University of Innsbruck Innsbruck Austria; ^4^ Division Clinical and Functional Anatomy Medical University of Innsbruck Innsbruck Austria; ^5^ Clinical Pharmacokinetics Unit Division of Intensive Care and Emergency Medicine Department of Internal Medicine I Medical University of Innsbruck Innsbruck Austria; ^6^ Department of Neurosurgery Medical University of Innsbruck Innsbruck Austria

**Keywords:** atherosclerosis, cholesterol ester transfer protein, HDL mediated cholesterol efflux capacity, in vivo reverse cholesterol transport, matcha green tea, New Zealand White rabbits, pulse wave velocity

## Abstract

**Introduction:**

Green tea is associated with decreased risk for cardiovascular disease and stroke. Matcha is a special kind of powdered green tea known for its use in the Japanese tea ceremony. Due to its influence on lipoprotein parameters, it has been postulated to exert antiatherogenic effects. This study investigates whether it modulates the high‐density lipoprotein (HDL) function and thereby influences the atherogenic process in an animal model with a strong influence on humans' situation.

**Methods and Results:**

After a pretreatment phase based on a standard diet, 10 female New Zealand White (NZW) rabbits are fed a high‐fat diet for 20 weeks. The treatment group is additionally administered 1% matcha during the whole experiment. Long‐term matcha treatment leads to lowered HDL cholesterol, impaired cholesterol transport manifested by reduced in vitro cholesterol efflux capacity, reduced cholesteryl ester transfer protein (CETP)‐mediated cholesterol ester (CE) transfer between HDL and triglyceride‐rich particles, and reduced macrophage‐specific in vivo transfer, where ian increased absorption of cholesterol in the liver but a decreased secretion into bile is observed. Pulse wave velocity, assessed by nuclear magnetic resonance, is increased in matcha‐treated animals, and a similar trend is observed for atherosclerotic lesion formation.

**Conclusion:**

Long‐term matcha green tea treatment of hypercholesterolemic rabbits cause impaired reverse cholesterol transport and increased vascular stiffness, and susceptibility for atherosclerotic lesion development.

## Graphical abstract

The reverse transport of cholesterol is responsible for the body's regulation of excess cholesterol. In rabbits, an animal model closest to the human situation in terms of lipoprotein metabolism, long‐term adjuvant therapy with matcha green tea alters this mechanism. The resulting metabolic changes are associated with atherosclerosis and exacerbate the clinical symptoms of cardiovascular disease.

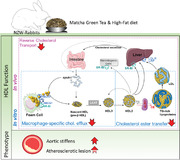

## 1 Introduction

Atherosclerosis is a chronic pathophysiological process that underlines the development of cardiovascular disease (CVD). Low levels of high‐density lipoprotein cholesterol (HDL‐C) have become well established as a robust independent risk marker for CVD patients.^[^ ^1^ ^]^ HDL particles have many potentially antiatherogenic properties including, antioxidative, anti‐inflammatory, antiapoptotic, and antithrombotic activities. They improve endothelial function, promote endothelial repair, increase angiogenesis, suppress monocytes and neutrophils' production and mobilization from bone marrow, and have anti‐diabetic properties.^[^ ^2^, ^3^, ^4^ ^]^

Interventional studies targeting elevation of HDL‐C with cholesterol ester transfer protein (CETP) inhibitors or niacin (on top of statins) had been stopped because they failed to show a beneficial effect on primary endpoints.^[^ ^5^ ^]^ These observations suggest other effects of HDL that HDL‐C does not readily reflect. A key function of HDL is the ability to promote the efflux of unesterified cholesterol from peripheral cells. The HDL‐mediated reverse cholesterol transport (RCT) is considered atheroprotective by transferring excess cholesterol back to the liver, where it is secreted into bile. Accordingly, new therapies are focusing on HDL functionality rather than simply on HDL‐C plasma concentrations.^[^ ^6^ ^]^ Despite notable research progress, CVD is the leading cause of death and disease worldwide, highlighting the need to identify new therapeutic strategies. Increased attention is directed towards nutraceuticals as possible supporting therapies and for primary prevention. Green tea polyphenol and catechins have beneficial and risk‐reducing effects for various chronic pathological conditions, including cancer, neurodegenerative diseases, diabetes, coronary heart disease, and CVD. These benefits have been linked to the antioxidant, antihyperlipidemic, anti‐inflammatory, antihypertensive, and antithrombotic properties of catechins.^[^ ^7^, ^8^, ^9^ ^]^

Matcha green tea is produced uniquely from leaves of shade‐grown tea trees. After harvesting and removing veins, stems, and impurities, it is immediately finely ground into a powder to prevent oxidation. With the shade's growth, this process yields an increased concentration of bioactive compounds, including amino acids and catechins. Therefore, it has been suggested that matcha contains more substances with potential health benefits than conventional green tea.^[^ ^10^ ^]^ The nutrient content of matcha green tea consists of 60–70% insoluble ingredients such as liposoluble vitamins, water‐insoluble dietary fibers, chlorophylls, and proteins and 30–40% of water‐soluble ingredients as polyphenols, caffeine, amino acids, water‐soluble vitamins, water‐soluble dietary fibers, saponin, and minerals.^[^ ^11^ ^]^ Drinking matcha green tea powdered leaves in the Japanese tea ceremony or mixing the powdered tea with food is considered particularly beneficial as the insoluble substances are also ingested.^[^ ^11^ ^]^

A protective role of green tea against CVD has been postulated since green tea interferes with micellar solubilization and absorption of cholesterol. Green tea activates AMP‐activated protein kinase and HMG‐CoA reductase inhibitor, stimulating lipogenesis and reducing endogenous cholesterol synthesis. Tea catechins have been reported to reduce the reabsorption of bile acids by inhibition of ileal apical sodium‐dependent bile acid transporter, to enhance the hepatic LDL receptor expression and the biliary excretion of cholesterol.^[^ ^12^ ^]^ Matcha tea was shown to protect mice on a high‐fat diet from hyperbolic triglyceride and total cholesterol levels.^[^ ^13^ ^]^ In Otsuka Long‐Evans Tokushima Fatty rats, the model for type 2 diabetes, matcha had protective effects against renal and hepatic damage by lowering glucose, triglyceride, and total cholesterol levels.^[^ ^14^ ^]^ However, rodents’ lipoprotein profile is different from that of humans, with HDL being the predominant lipoprotein particle, probably due to the lack of CETP in rodents’ plasma. Mice and rats are not prone to develop atherosclerosis. The lipoprotein profile of rabbits is more similar to that of humans than of rodents, as CETP mass and activity are present in rabbit plasma.^[^ ^15^ ^]^ Therefore, rabbits have crucial advantages as a model for diet‐induced lipid metabolic disorders leading to atherosclerosis in longitudinal studies, and due to their size, they allow repetitive blood sampling. We have previously used rabbits as a model to investigate lipid metabolism and atherosclerosis development induced by cholesterol‐enriched diets.^[^ ^16^ ^]^

In this report, we tested the effects of matcha green tea treatment on HDL function, reverse cholesterol transport (RCT), and atherosclerosis development in an animal model comparable to humans' situation.

## 2 Experimental Section

### 2.1 Animals and Experimental Design

Female New Zealand White (NZW) rabbits were purchased from Charles River Laboratories (Sulzfeld, Germany) and housed in flat deck cages in temperature (18°C) and humidity (50% RH) – controlled rooms with a 12 h / 12h light/dark cycle. At the age of 4 months, the average body weight was 3.2 ± 0.32 kg (mean ± SD), and rabbits were randomly divided into two groups of 5. There were no differences in body weight and cholesterol levels between the two groups. All experimental conditions were complied with the Austrian Animal Experimental Act (BGBI. I Nr. 114/2012) and approved by the National Committee for Animal Care of the Austrian Federal Ministry of Education, Science and Research (BMWF‐66.011/0110‐WF/V/3b/2015AQ6).

### 2.2 Diet and Experimental Design

**Figure 1:**
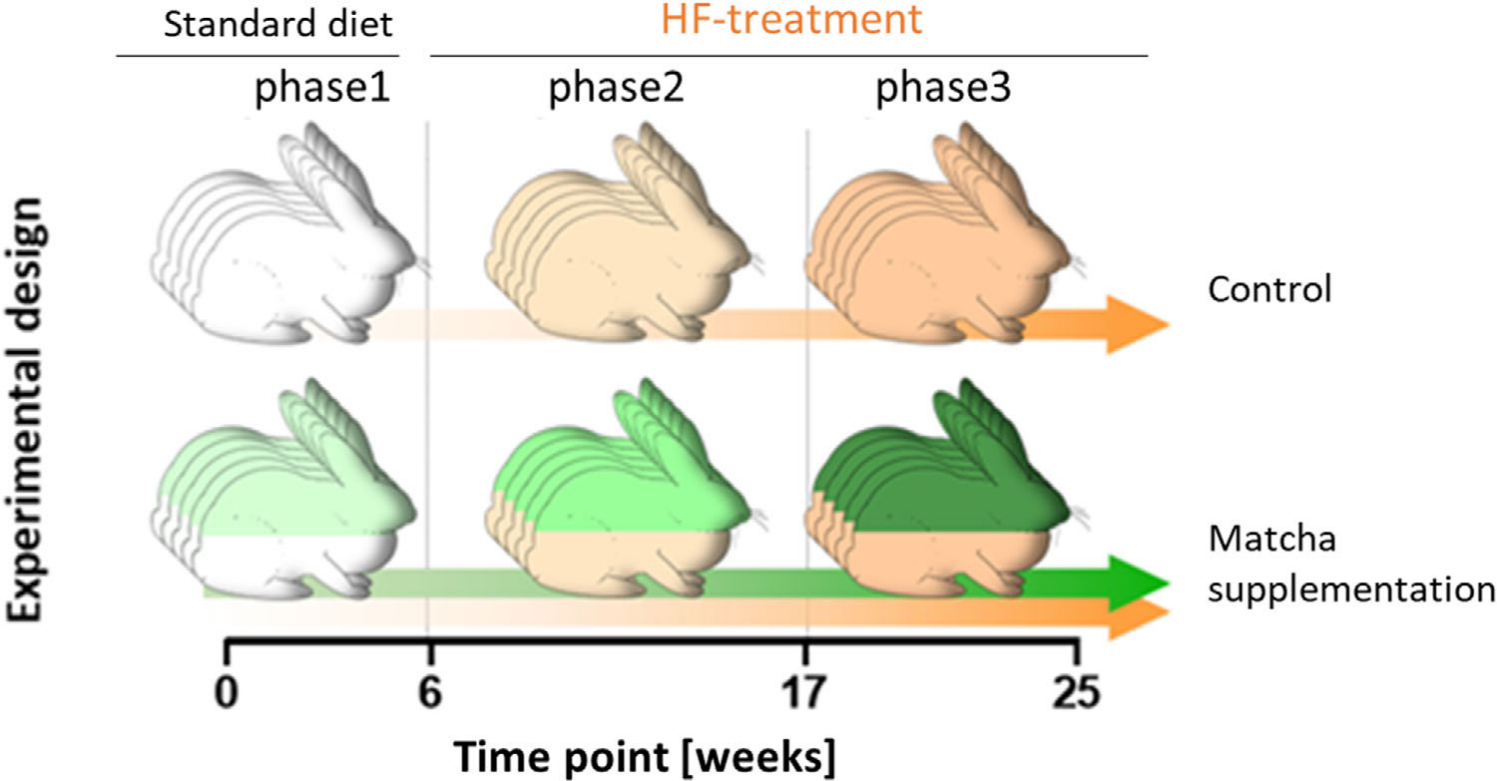
Experimental setup: Five female NZW rabbits were compared with five controls for 24 weeks. The experiment comprised three phases: phase1: 5 weeks of matcha green tea powder pretreatment versus standard rabbit diet; phase2: 10 weeks of HF‐treatment; phase3: further nine weeks HF‐treatment. The matcha group's animals received the same control rabbit diet for the entire 24 weeks, supplemented with matcha green tea powder (final concentration 1% w/w).

Rabbits daily obtained 120 g of standard diet V2333‐0 (Ssniff, Germany) for four weeks. Subsequently, the standard diet was enriched with the rabbit atherogenic diet containing 4.9% coconut oil, 1% cholesterol (w/w) E23113‐23 (Ssniff, Germany) to a final concentration of 0.15‐0.3% cholesterol for further 20 weeks. The test group also obtained a standard diet supplemented with matcha green tea (Sinas GmbH & Co.KG, Germany) to a final concentration of 1% during the whole 24 weeks (**Figure** **1**). The dosage equals 7.5 cups of tea for a human individual. Food consumption was monitored daily.

Blood sampling was performed weekly. Blood glucose was tested directly after sampling using an Accu‐Chek Performa Blood Glucose Meter (Roche, Germany).

### 2.3 High Performance‐liquid Chromatography (HPLC) for Caffeine Measurement

The stock solution of caffeine (FineTech Industry Limited, Lot No. 2‐MIT‐25‐1, purity 98%) was prepared in MeOH. Rabbit plasma samples 180 µL were spiked with ten µg (f.c.) caffeine (or an equal volume of MeOH) and mixed with 180 µL Acetonitrile (ACN), vortexed and centrifuged at 11 000 g for 5 min at 7 °C. The supernatant was collected.

The HPLC‐UV system (Thermo Fisher Scientific, Waltham) consisted of a degasser, UltiMate HPG‐3400RS, an autosampler WPS‐3000TPLR, a column oven TCC‐3000, and a UV–VIS detector VWD‐3400. The reversed‐phase column, a Zorbax Eclipse XDB‐C18 (150 mm × 2.1 mm with 5.0 µm particle size), was protected by a guard pre‐column a Zorbax 300SB‐C8 (2.1 mm × 12.5 mm), both by Agilent Technologies. The flow rate of the mobile phase was 1 mL min^–1^. The injection volume was 50 µL. Caffeine was detected at 230 nm.

The mobile phase A consisted of 0.2% (w/v) of formic acid in H_2_O adjusted to pH 5.0 with phosphoric acid (9.8%), mobile phase B was0.1%Formic acid in ACN. The linear gradient separation started with 0% of mobile phase B, which was increased to 5% within 8 min. Then, mobile phase B was increased to 25% and 100% within a further 2 and 7 min, respectively. Finally, the mobile phase was reduced to the initial concentration of 5% within another 6 min.

### 2.4 Liver Parameters and Antioxidant Capacity

The toxicity load on the liver was assessed in rabbit plasma samples using: ALP2 (Alkaline Phosphatase acc. to IFCC Gen. 2 ALP2S ‐ 0 333 3752 190), ASTLP (Aspartate aminotransferase acc. to IFFCC with Pyridoxal phosphate activation ‐ 4 467 493 190), ALTLP (Alanine Aminotranserferase acc. to IFCC with Pyridoxal phosphate activation ‐ 0 446 7388 190), GLDH3 (Glutamate Dehydrogenase Gen.3 ‐ 11 929 992 216), GGT‐2 (γ‐Glutamyltransferase ver.2 ‐ Standardized against IFCC ‐ 0 300 2721 122), LDHI2 (Lactate Dehydrogenase acc. To IFCC ver.2 ‐ 3 004 732 122), CK (Creatine Kinase ‐ 0 719 0794 190) (all by Roche Diagnostics), and canine CRP (Gentian VBR 0800).

Lipid peroxidation was measured using the thiobarbituric acid reactive substance (TBARS) assay detecting the level of MDA (malondialdehyde) in plasma samples according to manufacturer's instruction (Cayman Chemicals, 10 009 055).

### 2.5 Plasma Lipid Parameters and Lipoprotein Profiles

Blood samples were collected from the ear artery using S‐Monovette 9 mL, K3 EDTA (Sarstedt) using G22 needles. Plasma samples were collected and stored in aliquots at –80 °C. Total cholesterol, HDL‐C, and triglyceride concentrations were measured by colorimetric enzymatic diagnostic kits (Roche Cobas, Germany). LDL‐C and non‐HDL‐C concentrations were determined using the Friedewald Equation with an additional adjustment factor for high triglycerides.^[^ ^17^ ^]^

Lipoprotein profiles of each rabbit plasma sample were obtained by size fractionation with fast protein liquid chromatography (FPLC), as described previously.^[^ ^16^ ^]^ In brief, plasma samples were separated employing two Superose6 Increase 10/300 GL columns (GE Healthcare, UK) connected in series. Lipoproteins were eluted at a constant flow rate of 0.3 mL min^–1^ with PBS buffer. Total triglyceride and cholesterol content of individual fractions were determined by colorimetric enzymatic diagnostic kits (Roche Cobas, Germany). Lipoprotein curves were analyzed using spline interpolation.

### 2.6 In Vitro Cholesterol Efflux Capacity

J774.1A macrophage cells (ATCC#TIB‐67) were grown in DMEM supplemented with 10% fetal bovine serum as previously described.^[^ ^18^ ^]^ Macrophages were resuspended in a culture medium with 1% FBS and seeded into a 96‐well cell culture plate (50 000 cells per well) for the assay. Lipid loading was performed with 2.5 μCi ml^–1^ [^3^H]‐cholesterol. The cells were equilibrated in serum‐free growth media containing 0.2% (w/v) bovine serum albumin (essentially fatty acid‐free) for four h. After cAMP stimulation, an efflux medium containing 2.8% apoB–depleted serum was prepared by precipitating apoB‐containing lipoproteins with a mix of 8.2% tungstophosphoric acid hydrate and 6.2% 1 M MgCl_2_, is added for four h. All steps were performed in the presence of a 5 μg ml^–1^ acyl‐coenzyme A‐cholesterol acyltransferase inhibitor. The efflux of radioactive cholesterol from the cells was quantified by liquid scintillation counting. Samples were measured in triplicates. A serum‐free sample was used to determine passive diffusion of [^3^H]‐cholesterol and was treated as background. Three control samples were included in each plate to enable correction for inter‐assay variations. Relative CEC was determined as the percentage of control sample CEC in [%C] using the following formula: [(DPM in medium containing apoB‐depleted serum–mean DPM in acceptor‐free medium)/(DPM in control sample–mean DPM in acceptor‐free medium)]×100.

### 2.7 In Vivo Reverse Cholesterol Efflux Transfer

In vivo RCT analysis was performed following the protocol established by Dan Rader and colleagues^[^ ^19^ ^]^ and described by us earlier.^[^ ^20^ ^]^ Briefly, J774.1A macrophages were incubated with 2.5 mCi/ml [^3^H]‐cholesterol (PerkinElmer, USA) and 40 mg mL^–1^ acetylated LDL in DMEM containing 1 g L^–1^ glucose and 10% FBS for 48 h. The resulting foam cells were washed twice with PBS, equilibrated in serum‐free growth media containing 0.2% (w/v) BSA for 6 h, spun down, and resuspended in PBS. Typically, 3.5×107 cells containing 40×106 counts per minute [cpm] in 2.5 mL PBS were injected intraperitoneally into rabbits. Feces were collected at 24, 48 h, and plasma was collected at 6, 24, and 48 h post‐injection. Rabbits were sacrificed 48 h post‐injection by intravenous application of a pentobarbital overdose. According to Bligh and Dyer's procedure, tissue lipid extraction and fecal cholesterol extractions were performed.^[^ ^21^ ^]^ Radioactivity was assessed in plasma and lipid extracts of organs and feces by liquid scintillation counting. Liver, bile, and feces counts were expressed relative to plasma DPM µL^–1^ at 48 h.

### 2.8 Measurement of CE Transfer in Plasma

CETP‐mediated cholesterol ester (CE) transfer in total plasma was measured as plasma samples' capacity to promote the transfer of radiolabeled CE from exogenous HDL3 to apoB containing lipoproteins.^[^ ^22^ ^]^ In 50 µL plasma, 1.2 nmol [^3^H]‐CE‐HDL and 150 nmol Na‐iodoacetate were mixed with PBS containing 5% BSA to a final volume of 150 µL. Mixtures were incubated at 37 °C. The reaction was stopped by chilling the tubes on ice. Transfer reaction was linear up to 3 h (initial CE‐transfer rate) and reached its maximum after 16 h (total transfer of CE). ApoB‐containing lipoproteins were precipitated by adding 1% dextran sulfate and 0.5 M MgCl_2_. CE‐transfer was calculated as the total radiolabeled CE rate transferred to apoB‐containing lipoproteins compared to controls stored at 4 °C. Samples were measured in triplicates, and the results were expressed as the percentage decrease in [^3^H]‐CE in the supernatant.

### 2.9 Pulse Wave Velocity (PWV)

Magnetic resonance imaging (MRI) was performed on a 3 Tesla whole‐body MR scanner (Magnetom Skyra, Siemens, Erlangen, Germany). During measurements, rabbits were under general anesthesia and placed in a prone position on top of the integrated 32‐channel spine array receive coil. Anesthesia was performed by intramuscular injection of Ketamine (35 mg kg^–1^ body weight) combined with Xylazine (5 mg kg^–1^ body weight). An 18‐channel body array receiver coil was also placed over the rabbit to improve the signal‐to‐noise ratio. For PWV imaging, a velocity‐encoded phase‐contrast sequence was used as described earlier.^[^ ^23^, ^24^ ^]^ Flow velocity was encoded in three perpendicular directions, allowing the calculation of three velocity vector components and the flow velocity vector's magnitude. For all rabbits, velocity encoding was set to 150 cm s^–1^ and was adjusted if aliasing artifacts occurred. Retrospective ECG‐gating with 50 phases per cardiac cycle was applied. Rabbits had an RR‐interval of approximately 350 ms leading to a temporal resolution of about 7 ms. Velocity‐encoded images of the aorta were acquired in a transversal orientation perpendicular to the aorta at three slice positions: aortic arch, descending thoracic aorta, and abdominal aorta just above the origin of the renal arteries. Imaging parameters were as follows: TR = 25.92 ms; TE = 3.84 ms; flip angle:15 °, slice thickness = 8 mm; pixel size = 1.3×1.3 mm2; number of averages = 1; acceleration factor = 2; acquisition matrix: 256×192; FOV = 340 mm; percent phase FOV = 75; average scan time = 1 min 15 s per slice position.

For data analysis, the distance traveled between individual slices (Δs) was manually measured using ImageJ^[^ ^25^ ^]^ along the aortic luminal midline on an oblique sagittal view of the aorta. The regions of interest were manually placed on flow velocity‐encoded images to identify the aortic lumen within which velocity‐time curves over one RR interval were evaluated. The arrival time of the aortic pulse wave was defined as the half‐maximum between baseline and maximum flow velocity (t0.5) and calculated using a linear fit along with the systolic upstroke phase of the flow velocity. The transit‐time (Δt) between two image positions is calculated as the difference of the respective arrival times. Finally, PWV was calculated as the ratio of Δs /Δt (Figure 10).

### 2.10 Analysis of Atherosclerotic Lesions

Aortas were fixed in a 10% neutral buffered formaldehyde solution, and the extent of atherosclerosis was assessed by en face preparation and staining with Sudan IV (Roth, Germany). Lesion percentage of the whole intima was estimated using ImageJ 1.51n software (NIH, USA).

### 2.11 Statistical Analysis

The entire experiment lasted 24 weeks. We compared the test group (*n* = 5) with matcha pretreatment for 5 weeks to the control group (*n* = 5) with a standard diet.

Then we introduced HF‐treatment in all rabbits and compared the HF‐diet alone with the additional matcha treatment. We examined the short‐term (phase2 for 10 weeks) and long‐term effects (phase3, for further 9 weeks).

The samples examined in the three phases were calculated as the mean ± standard deviation, and the mean values in each of the three experimental phases were compared. Statistical analysis was performed in GraphPad Prism v.6 for Windows (GraphPad Software, USA). A two‐tailed unpaired Student's *t*‐test or Mann‐Whitney test was performed to determine statistical significance between the groups in the three experimental phases. Significance was set at *p* ≤ 0.05. The Grubbs test (alpha = 0.05) was performed to identify outliers that were subsequently removed from the analysis.

## 3 Results

### 3.1 Monitoring of Primary Parameters

**Figure 2:**
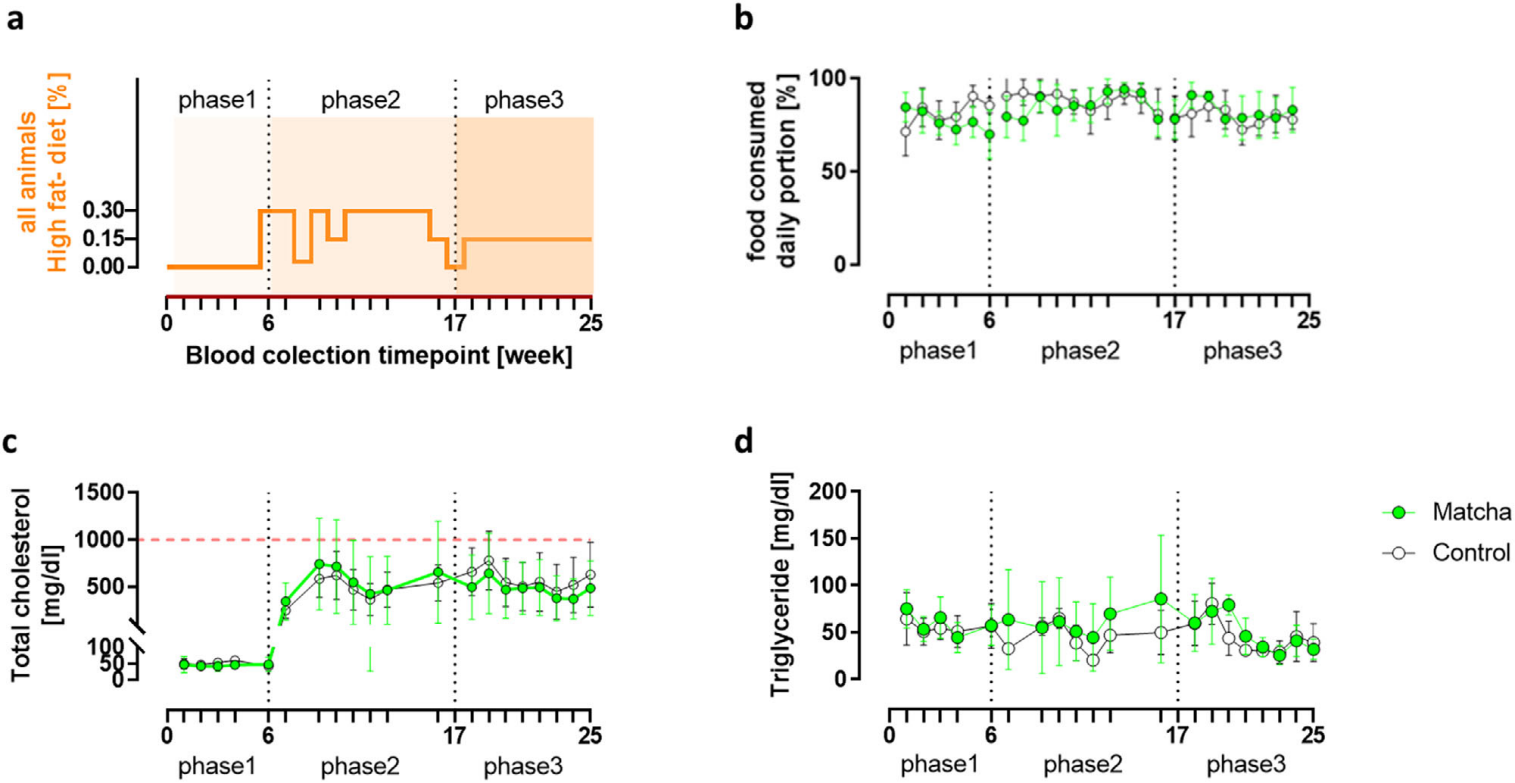
Experimental monitoring. A) All ten rabbits were given an HF‐diet from week 6 to week 25 (cholesterol was adjusted to keep the total plasma cholesterol level below 1000 mg dL^–1^). B) Food consumption means ±SD are given per week based on daily food monitoring. C) Total cholesterol and (D) total triglyceride plasma levels are represented as the mean ±SD, while the red dashed line represents the total cholesterol mark of 1000 mg dL^–1^. Dots represent distinct measurements at various time points of control (white dots) and matcha treated (green dots) animals. Ticks on the x‐axis indicate weekly blood draws.

The results from the high‐fat diet phase were divided into two phases (**Figure** **2**A). Total cholesterol and triglyceride plasma levels were monitored every week, and the total cholesterol was kept under 1000 mg dL^–1^ by adjusting the f.c. of the HF‐diet for all (Figure 2). Regarding food consumption, there were no differences between the two groups (Figure 2B).

**Figure 3:**
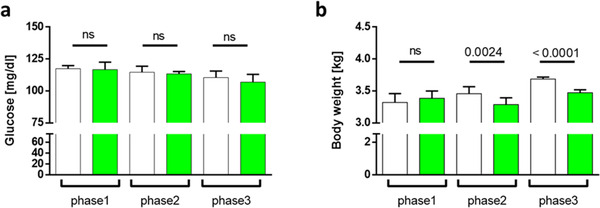
Matcha green tea causes a lower weight gain in rabbits with hyperlipidemia. A) Glucose levels at blood collection. B) Body weight was monitored weekly. The results are calculated as mean ±SD per experimental phase. White = controls; Green = matcha treated animals. Phase1 matcha pretreatment versus standard rabbit diet; phase2 (short‐term) and phase3 (long‐term) HF‐diet treatment versus additional matcha treatment.

While glucose levels decreased over the experiment, they did not differ significantly between groups (**Figure** **3**A). However, a long‐term matcha green tea diet resulted in significantly less weight gain when the HF‐diet was introduced than the control group (Figure 3B).

### 3.2 Matcha Treatment Reduced Oxidative Stress and did not Increase the Liver Toxicity Load

**Figure 4:**
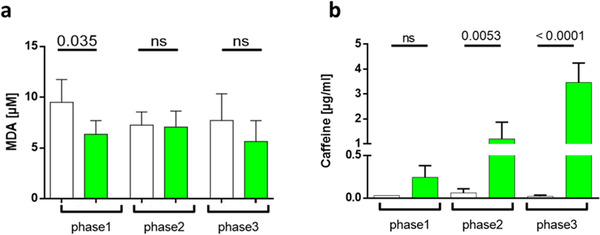
Effect of matcha green tea powder treatment on lipid peroxidation and caffeine content in rabbit plasma samples. Rabbits were treated with rabbit chow supplemented with matcha green tea (final concentration 1% w / w) and compared with controls. Plasma samples were assessed from phase1 (in week 6), matcha pretreatment against standard rabbit diet, as well as under HF‐treatment in phase2 (in week 16) and phase3 (in week 24). The results are presented as the mean ± SD. A) The TBARS assay determined malondialdehyde (MDA) values. B) Caffeine concentration peak areas were subjected to regression analysis. White = controls; Green = matcha treated animals. Phase1 matcha pretreatment versus standard rabbit diet; phase2 (short‐term) and phase3 (long‐term) HF‐diet treatment versus additional matcha treatment.

MDA's TBARS assay was evaluated as an estimate of lipid peroxidation. The pretreatment with matcha led to a significant decrease in MDA. While with a high‐fat diet, the trend was only seen after long‐term treatment (**Figure** **4**A).

We also compared the caffeine levels between the groups and confirmed caffeine levels' increase with the experiment's duration (Figure 4B). The calibration curve of caffeine using high‐performance liquid chromatography UV detection analysis had the regression equation y = 0.2256x + 0.0045 with a mean slope (SD): 0.2256, mean intercept 0.0045, and standard error 2.37E‐05. The detection limit (LOD) was 0.35 ng mL^–1^ and the limit of quantification (LOQ) was 1.05 ng mL^–1^.

**Figure 5:**
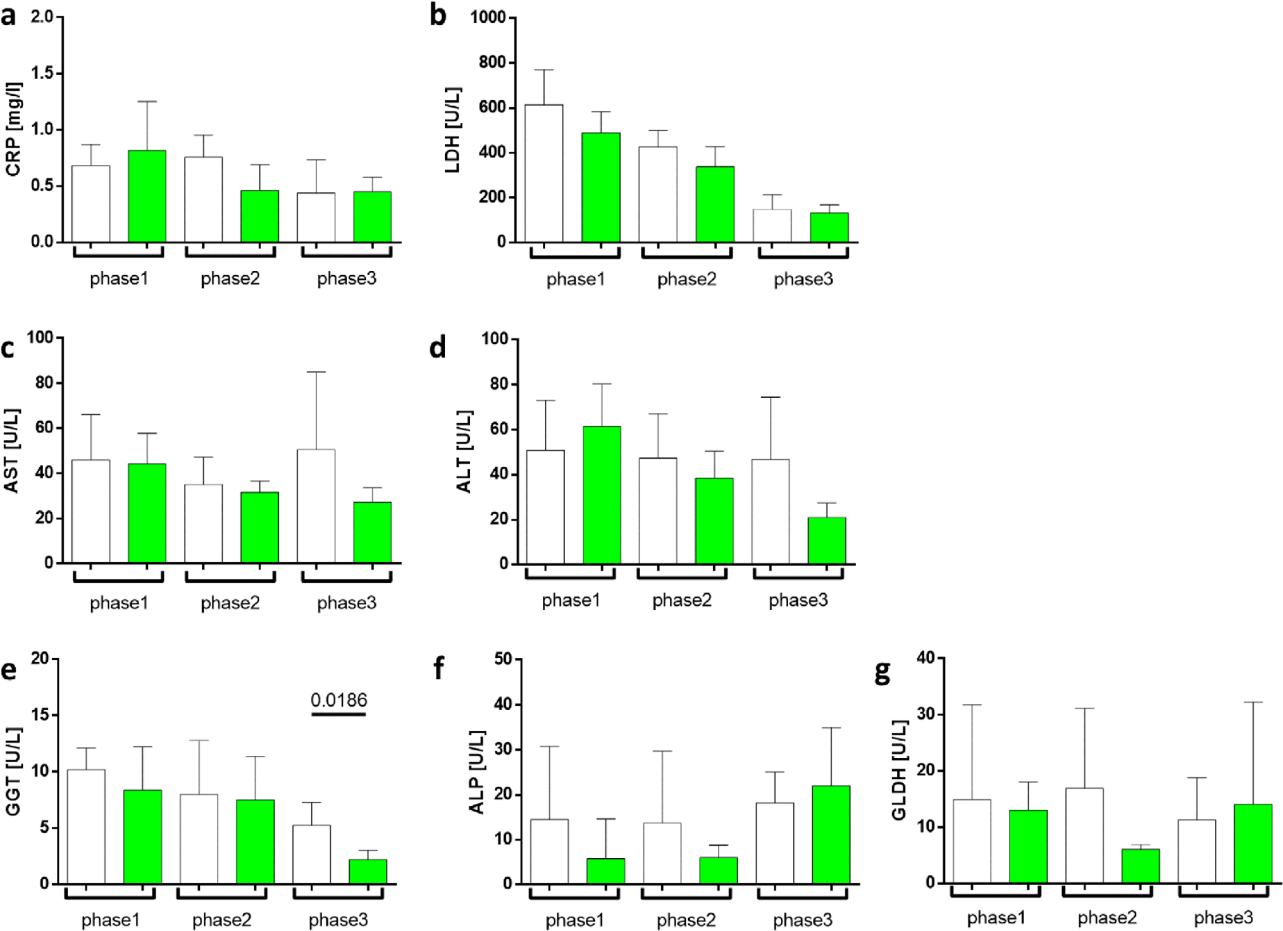
Liver function markers in matcha green tea treated rabbits and control rabbits. The following liver enzymes were assessed: (A) CRP; (B) LDH; (C) AST and (D) ALT; (E) GGT, (F) ALP, and (G) GLDH. The analysis was performed in pooled plasma samples (one per animal for each phase). Five control animals (white) and five matcha‐treated animals (green) are denoted as the mean ±SD.

Clinical parameters were determined to evaluate the toxicity load on the liver during all phases of the experiment. The following liver enzymes were assessed: marker for systemic inflammation C‐reactive protein (CRP); inflammatory and degenerative processes marker lactate dehydrogenase (LDH); liver injury markers alanine aminotransferase (AST) and aspartate aminotransferase (ALT); cholestasis markers gamma‐glutamyl transferase (GGT) and alkaline phosphatase (ALP); liver‐specific necrosis and hypoxic state marker glutamate dehydrogenase (GLDH). We did not observe any increase in liver parameters over the entire course of the experiment (**Figure** **5**).

### 3.3 Matcha Effects on Rabbit Blood Lipid Parameters and Lipoprotein Profile

**Figure 6:**
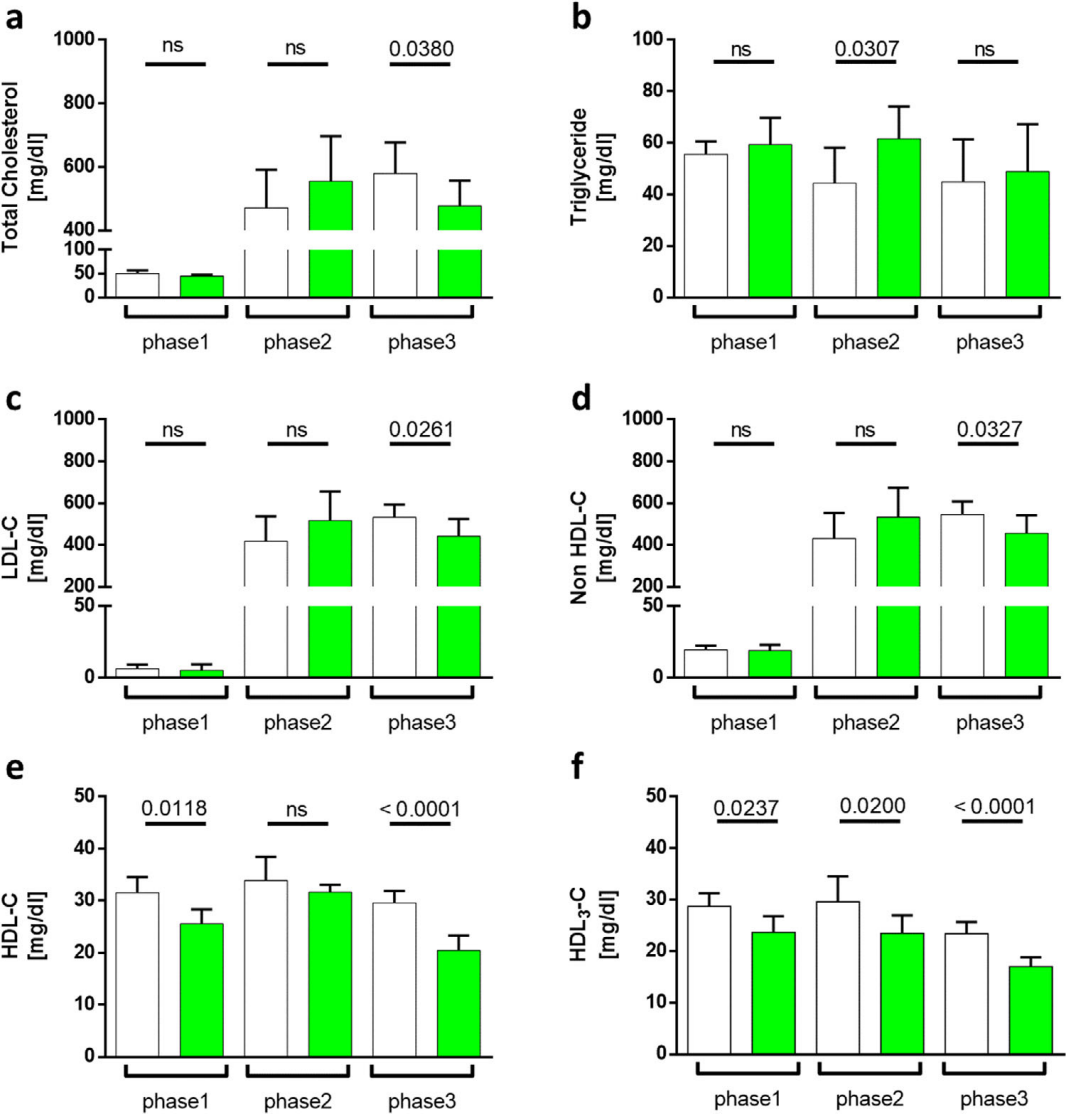
Matcha green tea treatment effect on basic plasma lipoprotein parameters. Matcha consumption effect on (A) total cholesterol levels compared to the control group, (B) total triglyceride, (C) LDL‐C, (D) non‐HDL‐C, and (E) HDL‐C levels. F) HDL3 cholesterol levels were obtained by PEG precipitation. The results are calculated as mean ±SD per experimental phase. White = controls; Green = matcha treated animals. Phase1 matcha pretreatment versus standard rabbit diet; phase2 (short‐term) and phase3 (long‐term) HF‐diet treatment versus additional matcha treatment.

Animals on HF‐treatment and long‐term matcha treatment showed significantly lower total cholesterol, LDL‐C, and HDL‐C concentrations. However, the matcha treatment did not affect triglyceride levels. In phase1 where animals are on a standard rabbit diet, matcha pretreatment caused a significant decrease of HDL‐C concentration. The matcha group had a lower concentration of small HDL (i.e., HDL3) in ApoB precipitated plasma than the control group (**Figure** **6**).

**Figure 7:**
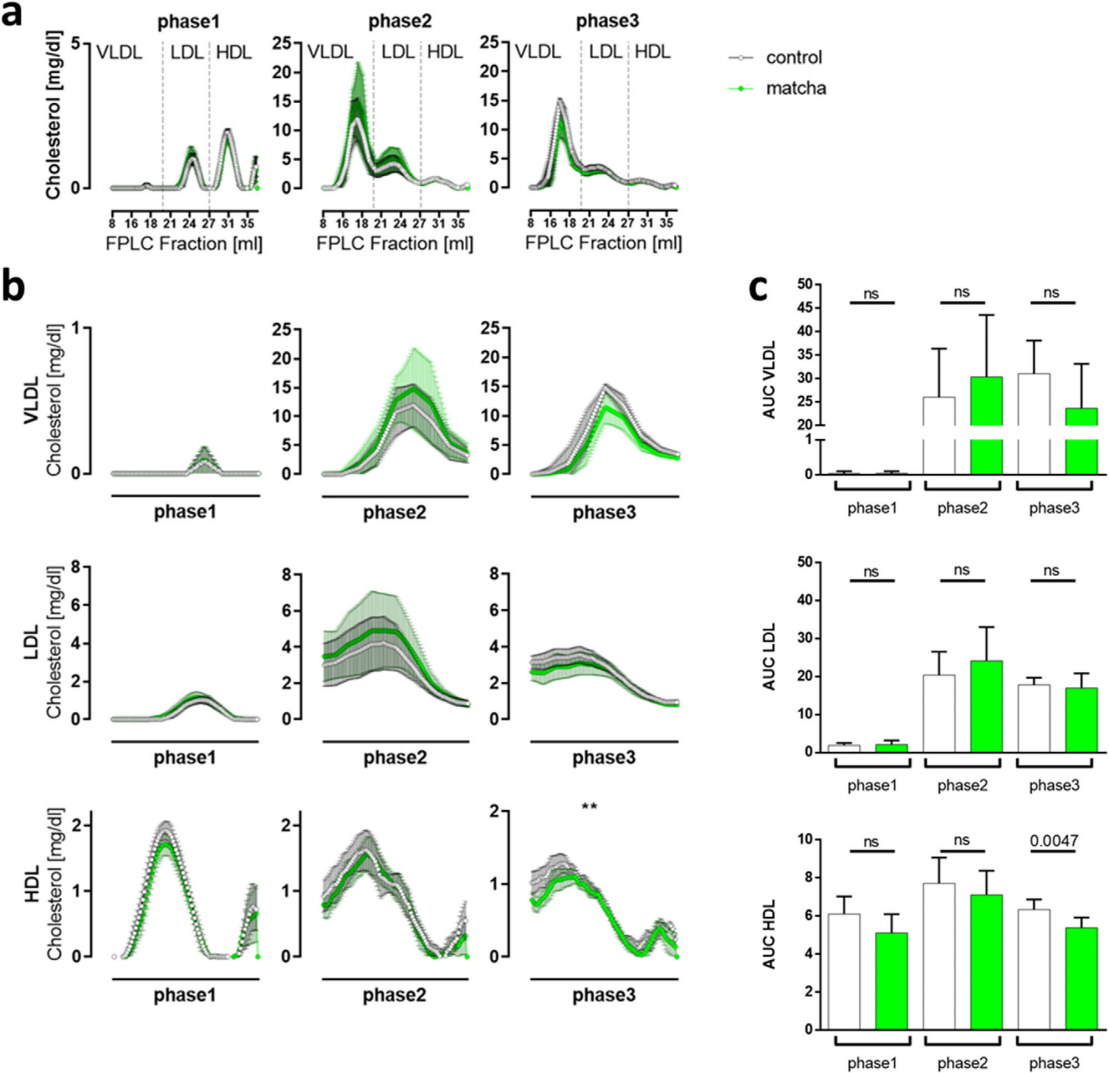
Characterization of rabbit lipoprotein profiles over three experimental phases. A) Plasma lipoproteins were separated into 80 fractions by FPLC, and their cholesterol content was measured. The lipoprotein profile curves were determined by spline interpolation. Per group, pooled plasma samples were used from five animals per experimental phase (replicates: phase1 *n* = 5; phase2 *n* = 7; phase3 *n* = 8) and shown as means ± SD. Rabbits received a standard rabbit diet (phase1), then HF‐diet for 10 weeks (phase2), and further HF‐diet for 8 weeks (phase3). The matcha rabbit group was additionally fed with matcha green tea. Fractions corresponding to VLDL, LDL, and HDL are marked. B) Lipoprotein profiles corresponding to VLDL, LDL, and HDL are shown separately and for each experimental phase as means ±SD. C) Quantification of lipoprotein species based on the area under the curve (AUC) for each time point. Dots represent means and the lines the ±SD. White = controls; Green = matcha treated animals.

Detailed lipoprotein profile analysis by FPLC showed that the observed effect on reduced plasma total cholesterol concentration in phase3 was mainly due to decreased HDL lipoprotein fraction. Overall, FPLC analysis of plasma samples confirmed the dyslipidemia of rabbits in phase2 and phase 3 on HF‐diet (**Figure** **7**A). Analysis of each lipoprotein's particle species’ interpolated splines showed no matcha treatment effect on total cholesterol levels during the pretreatment and early HF‐diet phases. Nevertheless, long‐term treatment resulted in lower cholesterol levels in phase3, *p* = 0.009 (Figure 7B,C). The total triglyceride in plasma did not differ significantly between the groups. We found increased levels of smaller HDL‐cholesterol particles in matcha‐treated rabbits within the late HF‐diet phase (Figure 7B).

### 3.4 The effect of matcha on HDL metabolism and HDL function

**Figure 8:**
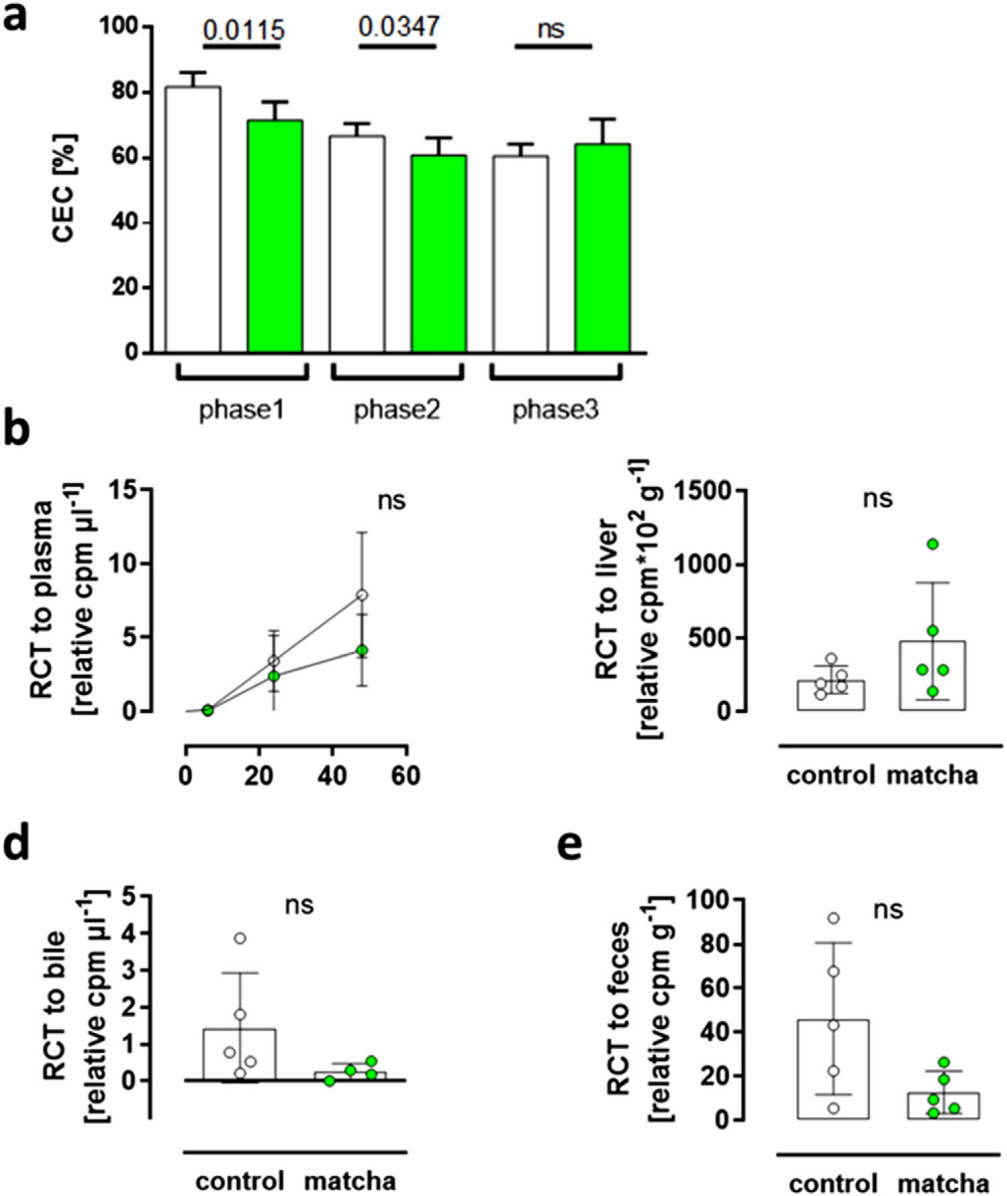
Matcha effect on in vitro CEC and in vivo RCT. A) Measurement of the CEC of plasma specimens from cholesterol‐laden J774 macrophages in vitro. The measurements were carried out in triplicate for each time point and each rabbit. Overall, the results are shown as means ± SD and the treatments were compared per experimental phase. Phase1 matcha pretreatment versus standard rabbit diet; phase2 (short‐term) and phase3 (long‐term) HF‐diet treatment versus additional matcha treatment. B) At the end of the experiment, macrophages’ reverse cholesterol transport was analyzed in vivo. [^3^H]‐cholesterol labeled J774 macrophages were injected intraperitoneally, and 48 h later, the^3^H‐tracer in plasma, liver and fecal sterols was measured. Dots represent RCT to plasma and bile in single, to the liver in triplicate, and feces in quadruplicate measurement per rabbit (*n* = 5). White = controls; Green = matcha treated animals.

Animals treated with matcha had a significantly lower in vitro CEC in phase 1, where the animals received a standard rabbit diet, and in phase 2 with the HF diet (**Figure** **8**A). The efflux capacity decreased in all animals after the introduction of HF‐diet. However, no differences were found within phase3 of the experiment. At the end of phase3, we measured the in vivo RCT, in which all NZW rabbits were injected intraperitoneally with [^3^H]‐cholesterol‐labeled macrophages. Forty‐eight hours post‐injection, we found a trend towards increased levels in [^3^H]‐cholesterol levels in the liver and decreased levels in bile and feces samples of matcha‐treated animals (Figure 8).

**Figure 9:**
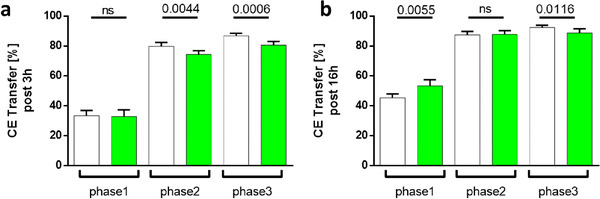
Measurement of CETP‐mediated CE transfer from HDL to the ApoB‐rich particles. (A) The initial and (B) the total rate of CE‐transfer were measured in plasma. The assay was performed in triplicates for each time point and each rabbit (phase1 *n* = 5; phase2 *n* = 7; phase3 *n* = 8 time points per rabbit). Results are presented by means ± SD of distinct measurements at various time points within the experimental phase of control (white dots) and matcha treated (green dots) animals.

CE‐Transfer assay, measuring the CE exchange between HDL particles and ApoB‐rich particles, showed decreased transfer rate at the onset (after 3 h) and of the total rate (after 16 h) of CE‐transfer in matcha treated animals (**Figure** **9**).

### 3.5 Matcha Treatment and Atherosclerosis

**Figure 10:**
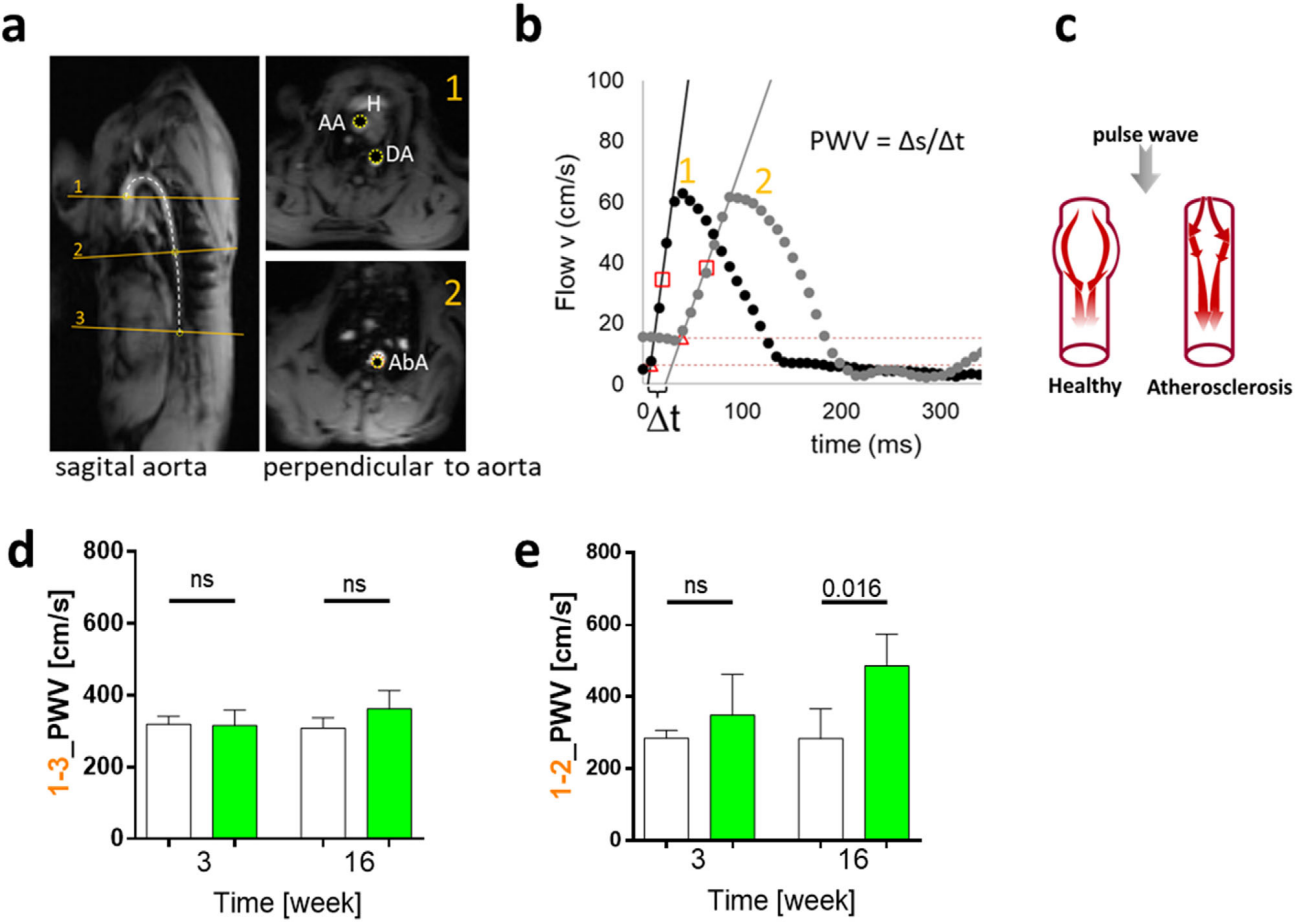
MRI‐based measurement of pulse wave velocity, PWV, and quantitative analysis of lesions within the aorta. A) Oblique sagittal view of the rabbit (H) heart and aorta in MRI with the three transversal cross‐sections along the aorta: (1) at the ascending part of the aortic arch (AA), (2) between descending aorta (DA), and abdominal aorta (AbA) and (3) the abdominal aorta just above the origin of the renal arteries. Distance between aortic planes was measured manually along the aortic luminal midline (white, dashed line). Transverse views perpendicular to the aorta show the defined regions where flow velocity was measured. B) Flow velocity‐time diagram of two cross‐sections derived from plane one at AA and plane two at AbA with best fit linear slope along the systolic upstroke. The arrival time of the pulse wave was defined as the time at half maximum from baseline to the peak of the systolic upstroke t0.5 (red square). The time difference between various slice positions was considered the transit‐time (Δt) of the pulse wave, and PWV was calculated as the ratio between ∆s and ∆t. C) A schematic representation where increased PWV or the increased speed of arterial pressure waves represents a tool for diagnosis and risk assessment of subclinical atherosclerosis. The measured PWV per rabbit at week three and week 16 was derived from plane one and plane 3 (D) and plane one and two (E), respectively.

The development of atherosclerosis was investigated by MRI‐based measurement of PWV, indicating aorta stiffness. No differences were observed within the pretreatment phase1. However, at the end of phase2, at week 16, matcha‐treated animals showed a significantly increased PWV (**Figure** **10**).

**Figure 11:**
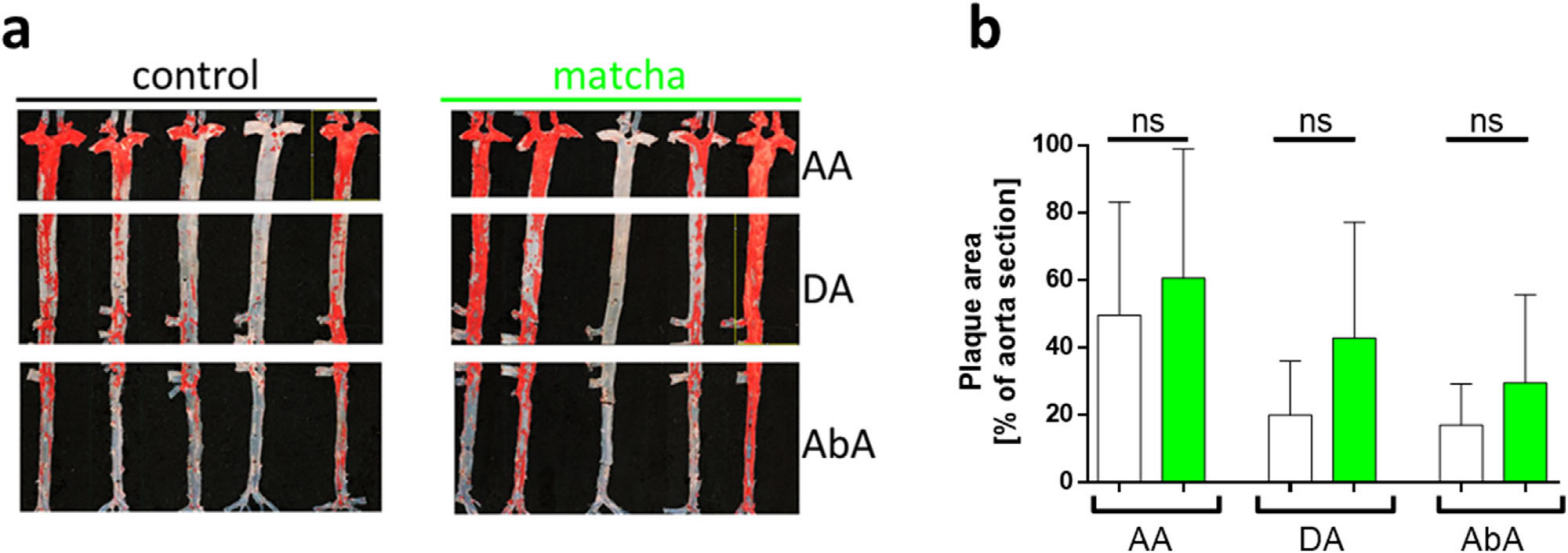
Analysis of the development of aortic lesions. A) En face analysis of the Sudan IV‐stained area of the entire aorta, represented by three areas: the aortic arch, AA; descending aorta, DA; abdominal aorta AbA. B) Quantitative representation of the percentage of the lesion in the total aortic area. Five control (white dots) and five matcha treated (green dots) animals are presented as means ± SD for three separate aortic areas.

At the end of the experiment, we analyzed the development of aortic lesions of all animals by Sudan IV staining. We observed a trend toward increased atherosclerosis lesions within the treatment group (**Figure** **11**).

## 4 Discussion

This report investigated the effect of matcha green tea treatment on HDL function regarding RCT, lipoprotein profiles, and development of atherosclerosis in an animal model comparable to humans' situation. Green tea catechins have been shown to exert protective and risk‐reducing effects in various chronic conditions.^[^ ^9^ ^]^ Matcha has higher catechin contents than other green teas, which was recently shown to manifest in a higher inhibition effect of reactive oxygen species production.^[^ ^26^ ^]^ Therefore, we decided to use this particular kind of green tea for our investigations.

Previous studies have shown that plasma cholesterol was affected only by high doses of green tea greater than 0.5% of the total diet.^[^ ^7^ ^]^ In the Japanese tea ceremony, 2–3 g of matcha is used per cup, corresponding to about 50 mg kg^–1^ for a human. We used a daily dosage of 1% matcha green tea (1.2 g), which would equal 7.5 cups of tea for a human individual. Epidemiological studies on green tea showed a serum cholesterol decrease in individuals drinking more than five cups of tea and a lower risk of all‐cause mortality and CVD mortality in patients consuming more than 7 cups day^–1^.^[^ ^27^, ^28^ ^]^

Pharmacokinetics in animal and human subjects showed that only a fraction of tea catechins could be absorbed.^[^ ^29^ ^]^ On the one hand, a prolonged and higher daily intake may mitigate poor bioavailability,^[^ ^30^ ^]^ and on the other, caffeine as a complementary bioactive improves the biological activity of catechins.^[^ ^31^, ^32^ ^]^ We assessed the protective role of matcha by measuring MDA levels in rabbit plasma samples. The biomarker for oxidative damage of lipids was reduced in pretreated animals and had a lower trend in matcha‐treated animals in phase3, indicating that matcha could have protective effects through modulation of free radicals. The caffeine content of matcha is relatively high compared to other green teas and could add to matcha's antioxidant potential.^[^ ^33^, ^34^ ^]^ We measured caffeine content in representative plasma samples stored at ‐80 °C and found significantly higher levels in phase2 and phase3. Therefore, the protective MDA level reduction in phase1 was not solely attributed to the caffeine content.

Animal toxicity studies indicated dose‐dependent hepatotoxicity of catechins.^[^ ^35^ ^]^ We measured concentrations of several blood liver markers to exclude liver‐related differences due to treatment, and we did not observe any elevation throughout the whole experiment. However, animals of the matcha‐treated group had a decreased weight gain over the experiment compared to control animals. Previous studies reported decreasing weight gain effects from tea polyphenols. This effect has been thought to be caused by carbohydrate‐inhibiting potency and microbiota modulations in the colon that affects short‐chain fatty acid production and improve lipid metabolism through AMP‐activated protein kinase activation.^[^ ^36^ ^]^

In a previous study in mice, the whole matcha tea showed higher effects of modulating hyperglycemia, dyslipidemia, and oxidative stress than the water‐soluble fraction of it alone. It is believed that water‐insoluble nutrients can play an essential role in lipid metabolism and antioxidant activity.^[^ ^13^ ^]^ Previous studies conducted in rabbits used drinking water enriched with brew infusions or extracts from green tea, respectively.^[^ ^37^, ^38^, ^39^ ^]^ Matcha green tea is actually consumed in water, but the tea powder itself is consumed. Our study used a solid diet enriched with complete matcha green tea powder to include the water‐insoluble nutrients of matcha green tea.

In previous reports, green tea treatment in rabbits did not affect lipid parameters or lowered total plasma cholesterol concentrations due to VLDL and LDL cholesterol reductions, respectively.^[^ ^37^, ^38^, ^39^ ^]^ We analyzed the lipoprotein profiles in our rabbits with high scrutiny by FPLC analysis and found that long‐term matcha treatment resulted in lower total cholesterol content, mainly due to a reduction in HDL‐C concentration. The HDL particle population also shifted towards a smaller mean particle size, which was in good agreement with earlier epidemiological studies showing that smaller HDL populations were associated with CHD in humans.^[^ ^40^ ^]^

We also examined various parameters related to HDL function. Matcha treatment reduced the CEC, which is the first and rate‐limiting step of RCT previously linked to CVD risk.^[^ ^2^ ^]^ In vivo RCT measurements reflect the contributions of diverse subcellular mechanisms of cholesterol mobilization, including ABCA1 and SR‐BI, respectively. We injected [^3^H]‐cholesterol‐enriched macrophages into our rabbits’ peritoneum to measure the flux of cholesterol into plasma, liver, and feces reflecting cholesterol mass movement in vivo.^[^ ^41^ ^]^ While we saw no difference in plasma cholesterol uptake, liver absorption was higher in matcha‐treated animals. The assay has some limitations in the rabbit model due to low concentrations of labeled cholesterol in the feces. While we measured low signals in animals treated with matcha, there was a tendency for more distinguished signals in the control group, which indicates an increased excretion of cholesterol in the bile for the control group. This hypocholesterolemic effect was also seen by Bursill et al., who suggested, as Brown et al., that green tea catechins do not alter the intestinal Acyl‐CoA activity: cholesterol acyltransferase (ACAT), which is rate‐limiting for intestinal esterification and absorption of cholesterol.^[^ ^39^ ^]^

CETP is a crucial enzyme that affects RCT. We were able to investigate this issue, as in contrast to rodents, rabbits express CETP in their plasma. Accordingly, we found a decreased CE‐transfer rate in matcha‐treated rabbits pointing towards a slower RCT, which is in good agreement with our in vitro and in vivo cholesterol efflux measurements, respectively.

Finally, the development and extent of atherosclerosis were assessed by noninvasive measurements and quantification of aortic lesions. Vascular stiffens is an early marker for atherosclerotic changes. MRI‐based measurements of PWV were performed in phase1 and phase2 of the experiment. PWV is the wave's speed caused by the heart's contraction that propagates through the body in the great arteries' wall and depends on the vessels’ elasticity. At the aortic arch (where the plaque formation usually starts in rabbits), we observed an increased PWV in the matcha‐treated group. Indeed, the matcha‐treated group's plaque formation at the end of the experiment revealed the same trend as the PWV.

The primary study limitation is the number of animals included due to the type of animal system. In our matcha‐treated group, we had a so‐called hypo‐responder animal that consumed the same amount of the daily portion but did not show elevated cholesterol levels. This observation has been reported by Overturf et al., in 1989, possibly due to the enhanced production and secretion of bile acids mediated by high expression of 7α‐hydroxylase.^[^ ^15^ ^]^ However, we were able to find significant effects of matcha treatment in our experiments. Also, the in vivo RCT assay was developed in mice and may not be directly applicable to the rabbit system. The tracer measurements in feces are limited due to the high content of cellulose, which leads to reduced sensitivity. Even the recommended addition of sodium hypochlorite did not improve our results.^[^ ^42^ ^]^ Nevertheless, we were able to detect subtle differences between matcha‐treated animals and controls.

One of the strengths of this work is using an animal system with a high impact on the human situation. The lipoprotein metabolism of rabbits is similar to humans in many respects but differs from rats and mice.^[^ ^15^ ^]^ For example:

-  rabbits express CETP mass and activity in plasma;
-  they are LDL‐mammals like humans and produce similar apoB‐containing lipoproteins in the liver like those observed in humans;
-  rabbits are sensitive to cholesterol diet;
-  their lipoprotein profile is more similar to that of humans.

Therefore, they are a valid model to study lipid metabolism and atherosclerosis development induced by cholesterol‐enriched diets without pathological predisposition.^[^ ^43^ ^]^ We used female rabbits because they have slightly higher total cholesterol levels and could reach atherosclerotic levels faster subjected to an atherogenic western diet. In addition, female rabbits show no difference in LDL levels and, with later atherosclerosis, develop a more significant lesion burden in the descending aorta than do age‐matched males.^[^ ^15^, ^44^, ^45^ ^]^

To our knowledge, this is the first report on matcha green tea's effects on HDL metabolism. Additionally, we investigated all green tea compounds' effects using the whole matcha tea powder instead of certain extracts. Finally, we kept total cholesterol plasma levels under 1000 mg/dl preventing liver toxicity effects. Weekly blood collections and noninvasive PWV measurements enabled us to evaluate long‐term as well as short‐term treatment phases.

## 5 Conclusions

The intervention study showed that treatment with matcha green tea powder, at least in rabbits, not only induces metabolic changes known to be associated with the development of atherosclerosis but even aggravated the clinical symptoms of CVD.

We can report two key findings: First, based on these results, long‐term matcha green tea treatment may no longer be viewed solely as atheroprotective. Second, the therapeutic modulation that affected HDL function related to RCT was associated with atherosclerosis formation and progression.

In conclusion, HDL‐mediated reverse cholesterol transport is related to atherosclerosis development and therefore has implications for being a diagnostic marker and therapeutic target.

## Conflict of Interest

The authors declare no conflict of interest.

## Author Contributions

M.H. was involved in conceptualization, data curation, methodology, project administration, validation, visualization, writing—original draft preparation, and writing—review and editing. C.S. performed experiments and analyzed data. C.K. established and performed a PWV experiment; supported data analysis and interpretation. B.M. performed plaque phenotype analysis. R.W. provided expertise on the analysis and interpretation of HPLC data. C.K. offered technical support. A.B. and D.S.M. contributed to the methodology, material support and writing—review and editing. A.R. conceived, designed, and supervised the project, performed experiments, and critically revised the manuscript.

## Data Availability

The data that support the findings of this study are available from the corresponding author upon reasonable request.
